# Annotations

**Published:** 1903-10-17

**Authors:** 


### Annotations.

### Maternal Responsibility.

The Leicester Guardians have had put before them a suggestion which, if carried into law, would make the position of a mother so difficult that only the utterly thoughtless would be willing to fill it. It was that a mother should be held responsible for the fall of her daughter, if the latter was under 16 years of age, and that for the daughter's fault the mother should be punished, if need be, by imprisonment. Happily for their reputation for common sense, the ladies' committee of the Board, to whom the proposal was handed for consideration, refused to sanction the idea. As they pointed out, when a girl begins to work outside the home, her mother's influence is by no means all supreme. She has begun an independent life, and has her own ideas. As a matter of fact, the influence of girl-companions and fellow-workers is often much more powerful than that of the mother. Even when a girl does not work outside the home, it does not follow that her mother's example and precept have supreme weight with her. She is affected by her whole environment, of which her mother is part, and perhaps an important part, but not by any means the whole. Moreover, girls of the working class feel themselves grown up while they are still in their early teens, and thus are open to a woman's temptations before they have a woman's wisdom. The whole problem is as difficult as it is painful, but it would be unjust to cast the whole blame of it upon the girls' mothers, and it is not for poor humanity, so liable to err as it is, to enter upon the difficult task of settling vicarious responsibility and apportioning vicarious punishment. And the problem seems really to be in process of solution, even if the progress be slow. Mr. Charles Booth's last volume tells us that boy-and-girl marriages are becoming less numerous, and if this be so we may be sure that those relations which often lead to forced marriages are becoming also less common. Evening schools for the serious and clubs of various kinds for the more frivolous, are arousing new interests in the young of both sexes, and these wider interests help to relegate to a lower place the otherwise supreme attraction of the "young man" and the "best girl." In such influences let us put our trust, and not seek to hurry reform by the punishment of unhappy mothers, to whom their daughters' fall is humiliation enough, without the addition of public trial and imprisonment.

### Disinfection Difficulties at Liverpool.

The report of Dr. E. W. Hope, the Medical Officer of Health for Liverpool, shows, among other things, the difficulties under which the sanitary authorities labour when they are trying to do their best for those under their charge. In cases of infectious disease, it is often necessary to destroy clothing and bedding. Formerly it was the custom to give those from whom these things were taken compensation for their loss in money. But it was often found that the money thus received was spent in drink, instead of being put to its proper use, and thus the people were left without clothing. To remedy this the sanitary authorities now keep at the depot a supply of mattresses, bedding, etc., which are given to the poor whose things have been destroyed owing to being infected. This storing of mattresses and blankets seems to us to be rather a clumsy method of coping with the difficulties of the situation, as the demands on the store must be very variable, and there is always the risk that if the supply is large and the demand small, the goods may suffer from moth or damp. It has been found necessary for the disinfecting staff to undertake the duty of stripping the wall paper from infected rooms, as there were often delays and difficulties in compelling the owners of the property to do it. Before being stripped, the paper is sprayed with a solution of perchloride of mercury, and after it is taken from the walls it is conveyed in sacks, kept specially for that purpose, to the refuse destructor, where it is promptly burnt. Another very sensible precaution is the removal of books belonging to lending libraries, when they are found in infected houses, and either disinfecting or destroying them. The thoughtlessness of some people with regard to library books is remarkable. Books to amuse the invalid or his nurse are borrowed and returned, without a thought of the possible infection which they may carry to the next reader. It is a safe rule to warn the occupants of every house where there is a case of infectious disease, that books or toys which have once gone into the sick room must never come out again.

### Rheumatic Iritis.

It has been very confidently maintained in recent years that such a condition as rheumatic iritis does not exist. Cases so named, it has been said, are the results not of rheumatism but of gonorrhœa. It is obvious that such a statement is a very serious one, and its application in individual instances is capable of producing much personal and domestic unhappiness. The dogmatic precision of the doctrine is well calculated to impress the inexperienced who, as is inevitable in early clinical training, see many of the postponed penalties of venereal disease. And the somewhat loose application of the term "rheumatic" in various other directions has facilitated the success of the assault upon the older view in reference to iritis as one of the possible results of rheumatism. Clinical evidence nowadays is somewhat under a cloud, and the demand is for chemical and bacteriological demonstration. But even those who make this demand must now agree that iritis may be purely and solely rheumatic in origin and nature. For Payne and Poynton in their investigation into the bacteriology of rheumatic fever have noted that occasionally the intravenous injection into rabbits of a minute diplococcus isolated from the blood, synovial fluid, etc., of patients suffering from rheumatic fever is followed not only by effusion into the joints and heart disease but also by irido-cyclitis. This experimental result is therefore an exact demonstration of the accuracy of the older clinical teaching, and apart from its interest is of practical social significance. If it be urged that in a given case it is not justified to label a case as "rheumatic" iritis without demonstration of the specific organism, it must be remembered that this principle is equally applicable to a diagnosis of "gonorrhœal" iritis. Further, it may be reasonably maintained that iritis may be an isolated event and yet be an evidence of rheumatism. It is not possible, perhaps, to produce conclusive proof of this proposition, but as such events as tonsillitis, chorea, endocarditis, etc., occur apart from the more conventional phenomena of rheumatism it is a natural conclusion that iritis may stand in the same position.

